# Efficacy and hemodynamic safety of perioperative dexmedetomidine for the prevention of postoperative delirium in elderly patients undergoing cardiac surgery: an updated systematic review and meta-analysis of randomized controlled trials

**DOI:** 10.3389/fneur.2026.1895312

**Published:** 2026-08-12

**Authors:** Anan Li, Haopeng Wu, Kai Zhang, Jianzhi Ying

**Affiliations:** 1 Department of thoracic surgery,The First People’s Hospital of Taizhou, Taizhou, China; 2 Department of Emergency Medicine, The First People’s Hospital of Taizhou, Taizhou, China; 3 Department of Critical Care Medicine, Second Affiliated Hospital, Zhejiang University School of Medicine, Hangzhou, China

**Keywords:** cardiac surgery, dexmedetomidine, elderly, meta-analysis, postoperative delirium

## Abstract

**Background:**

Postoperative delirium (POD) is a common and clinically important complication after cardiac surgery in older adults. We performed an updated systematic review and meta-analysis of randomized controlled trials (RCTs) to evaluate the efficacy and hemodynamic safety of perioperative dexmedetomidine for POD prevention in older adults undergoing cardiac surgery.

**Methods:**

We searched PubMed, Embase, Scopus, and the Cochrane Central Register of Controlled Trials from inception through July 12th, 2026 for RCTs comparing perioperative dexmedetomidine with placebo or alternative sedative /analgesic regimens in elderly patients aged 60 years or older, or in extractable elderly subgroups, undergoing cardiac surgery. Primary outcomes were POD incidence and delirium duration. Secondary outcomes included length of intensive care unit (ICU) stay, length of hospital stay, and the risk of bradycardia and hypotension.

**Results:**

Fifteen RCTs involving 3,274 patients were included. Perioperative dexmedetomidine was associated with a lower incidence of POD (Risk Ratio [RR] 0.70, 95% Confidence Interval [CI] 0.56 to 0.86, *p* = 0.001, I^2^ = 19%; GRADE: moderate certainty) and shorter duration of delirium among patients with POD (Mean Difference [MD] −1.12 days, 95% CI −1.89 to −0.35, *p* = 0.01, I^2^ = 83%; GRADE: low certainty). The apparent benefit was more evident in active-comparator trials than in placebo/saline-controlled trials, but this comparator-dependent pattern should not be interpreted as proof of a uniform neuroprotective effect. However, dexmedetomidine was associated with increased bradycardia (RR 2.08, 95% CI 1.31 to3.31, *p* = 0.0019, I^2^ = 0%; GRADE: low certainty). No significant differences were observed for length of ICU stay, length of hospital stay, or hypotension.

**Conclusion:**

Perioperative dexmedetomidine may reduce POD incidence in selected older cardiac surgery patients, particularly when compared with selected active sedative or analgesic regimens. However, the certainty of evidence was moderate, and the effect was less clear in saline/placebo-controlled trials. Evidence for delirium duration was based on few affected patients, showed substantial heterogeneity, and should not drive the main clinical conclusion. Dexmedetomidine increased reported bradycardia, and clinical use should be individualized with careful hemodynamic monitoring.

**Systematic review registration:**

https://osf.io/f6nwu

## Introduction

Approximately 1.5 million cardiac surgeries are performed worldwide annually, and older adults comprise a growing proportion of this population (1). Postoperative delirium (POD) is an acute disturbance of attention and cognition that occurs frequently after cardiac surgery and is associated with longer hospitalization, functional decline, institutionalization, and mortality (2, 3). Advanced age is a strong predisposing factor for POD, reflecting reduced physiologic reserve, higher comorbidity burden, and greater vulnerability to perioperative stressors (4, 5). Dexmedetomidine, a selective alpha-2 agonist, provides sedation and sympatholysis with limited respiratory depression and may reduce exposure to some sedative regimens associated with delirium risk (6). However, dexmedetomidine can cause clinically relevant bradycardia and hypotension, particularly in older cardiac surgical patients with conduction disease, beta-blocker exposure, or limited hemodynamic reserve.

Several recent meta-analyses including trial sequential analysis and Bayesian approaches have evaluated dexmedetomidine after cardiac surgery (7–10). These reviews generally suggest a possible reduction in POD but also emphasize uncertainty from heterogeneity, publication bias, trial limitations, and bradycardia risk. Most prior reviews addressed broader adult cardiac surgery populations rather than randomized evidence restricted to older patients. The present review therefore has a narrower focus: elderly cardiac surgery patients, outcome-specific data availability, comparator-centered interpretation, and formal certainty grading for delirium and hemodynamic outcomes.

## Methods

This meta-analysis followed the updated Preferred Reporting Items for Systematic Reviews and Meta-Analyses (PRISMA) guidelines (Supplementary material 1). The protocol was registered on the Open Science Framework,^1^ protocol amendments are reported in the revision history and Supplementary material 2. We searched PubMed, Embase, Scopus, and Cochrane Library for relevant studies through July 12th, 2026. The database-specific search strategies are provided in Supplementary material 2.

### Inclusion and exclusion criteria

Studies were included if they met the following criteria: (1) Population: elderly patients (defined as trials in which all randomized participants were aged 60 years or older or trials from which age-specific data for patients aged 60 years or older could be extracted) undergoing cardiac surgery (coronary artery bypass graft [CABG], valve surgery, valve replacement, aortic surgery, or combined CABG and valve replacement); (2) Intervention: perioperative intravenous dexmedetomidine at any dose, timing, or duration; (3) Comparison: intravenous placebo/saline or alternative sedative/analgesic regimens at any dose, timing, or duration; (4) Outcomes: primary outcomes were the incidence of POD (assessed using validated diagnostic instruments) and delirium duration. Secondary outcomes included length of ICU stay, length of hospital stay, and incidence of bradycardia and hypotension; (5) Study design: randomized controlled trials (RCTs); (6) Language: english only. Trials in which dexmedetomidine was embedded in a combined sedation strategy without an isolatable dexmedetomidine-versus-control comparison were excluded from the final quantitative synthesis.

We excluded trials where the clinical data for the elderly subgroup (age ≥ 60 years) could not be isolated, trials conducted on pediatric populations, observational studies, case reports, animal studies, conference abstracts, and non-peer-reviewed preprints.

### Study selection

Search results were imported into EndNote to remove duplicates. Two authors independently screened titles and abstracts of potentially eligible studies, then retrieved and assessed full texts for eligibility. Disputes were resolved through discussion with a third adjudicator.

### Data extraction and quality assessment

Two authors independently extracted authorship, publication year, study design, randomized sample size, population demographics, intervention and control methods, intervention duration, and delirium assessment tools using a standardized form adapted from the Cochrane Data Collection template. When studies reported continuous outcomes as medians and interquartile ranges (IQR), we converted these to means and standard deviations (SD) using the methodology of Wan et al. (11). For multi-arm or factorial trials, eligible dexmedetomidine and control arms were selected according to the prespecified comparison, and shared control groups were not double counted.

**Figure 1:**
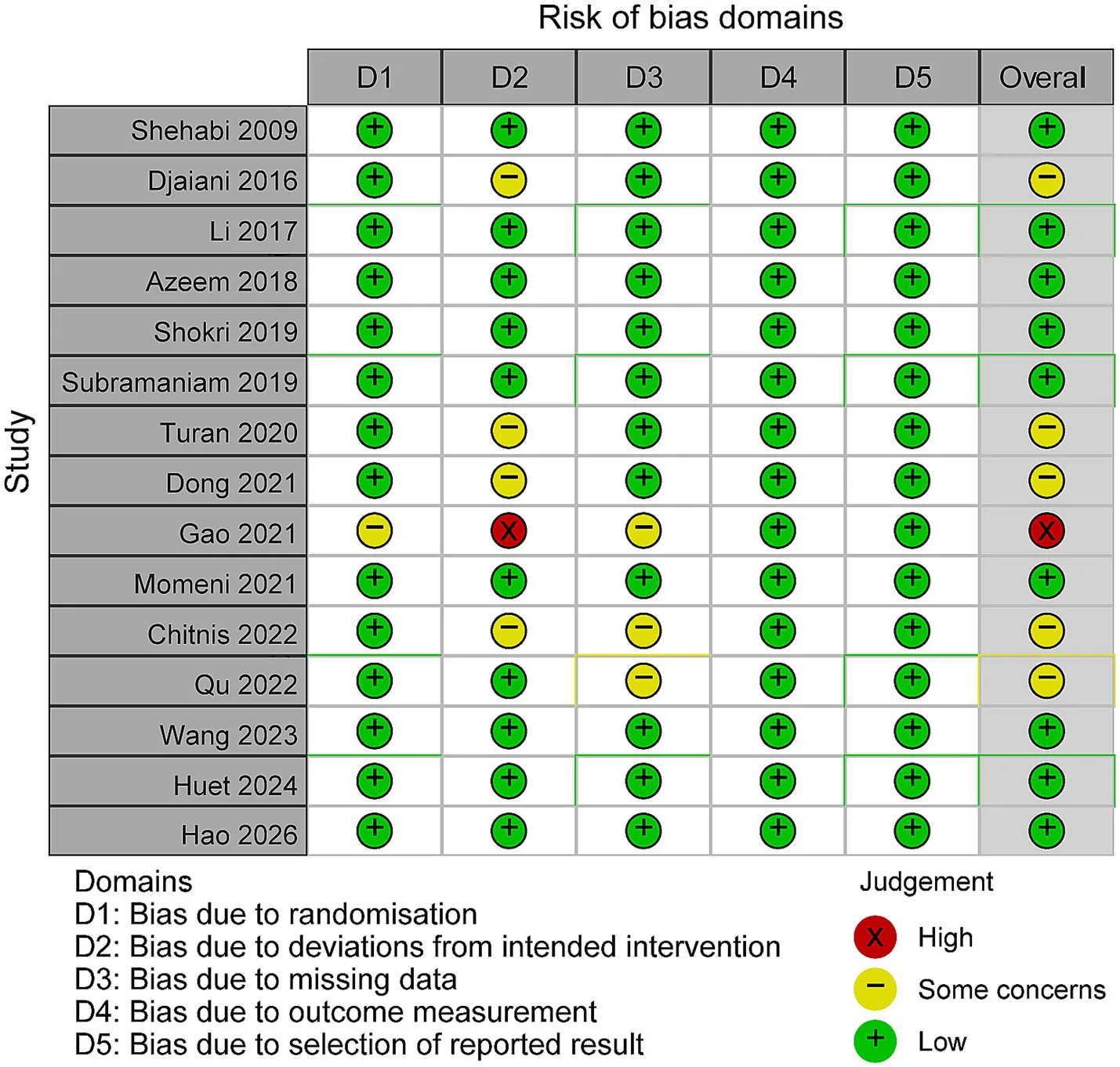
Risk of bias 2 of all included studies.

Methodological quality was independently assessed by two authors using the Cochrane Risk of Bias tool (RoB 2) (12). RoB 2 judgments were made at the outcome level (Supplementary material 3), and the Figure 1 summarizes the overall judgment across assessed outcomes for each study. Disagreements were resolved by consensus with a third adjudicator. Certainty of evidence for each main outcome was assessed using the GRADE framework, considering risk of bias, inconsistency, indirectness, imprecision, and publication bias (13).

### Statistical synthesis and analysis

All statistical computations and visualizations were performed using R version 4.4.2. The “meta” package was used for meta-analysis, and the “robvis” package was used for risk-of-bias visualization (14, 15). Dichotomous data were pooled as risk ratios (RRs) with 95% confidence intervals (CI) using a Mantel–Haenszel model. Continuous data were pooled as mean differences (MDs) with 95% CI using an inverse variance model. Given the clinical and methodological heterogeneity across trials, including variation in dexmedetomidine dose, timing, duration, and comparator regimen, random-effects models were used for the primary analyses. Between-study heterogeneity was quantified using the Higgins I^2^ statistic (16), with approximate thresholds of 25, 50, and 75% indicating low, moderate, and substantial heterogeneity, respectively. Publication bias was assessed using funnel plots and Egger’s regression test; when detected, the trim-and-fill method was applied to calculate corrected estimates. However, the funnel plots, Egger tests, and trim-and-fill analyses were interpreted only as exploratory for outcomes with fewer than 10 studies.

Comparator type was prespecified as a key analytic and interpretive factor because placebo/saline-controlled trials and active-comparator trials answer different clinical questions. We therefore summarized dexmedetomidine versus placebo/saline and dexmedetomidine versus active sedative or analgesic regimens separately, in addition to reporting the overall pooled estimate as a broad summary. Subgroup analysis was stratified by intervention duration (≤ 24 h versus > 24 h). Tests for subgroup differences were used where data were available; subgroup findings were interpreted cautiously, especially when interaction tests were not statistically significant or when few trials contributed to a subgroup.

Sensitivity analysis assessed individual study impact through sequential exclusion. For length of stay outcomes, we also performed a sensitivity analysis excluding studies in which medians and IQRs were converted to means and SDs. Moreover, random-effects meta-analyses were additionally performed using the Hartung-Knapp-Sidik-Jonkman adjustment to account for uncertainty in the estimation of between-study variance, particularly when the number of studies was small or heterogeneity was present (17).

## Results

### Study selection and characteristics of included studies

**Figure 2:**
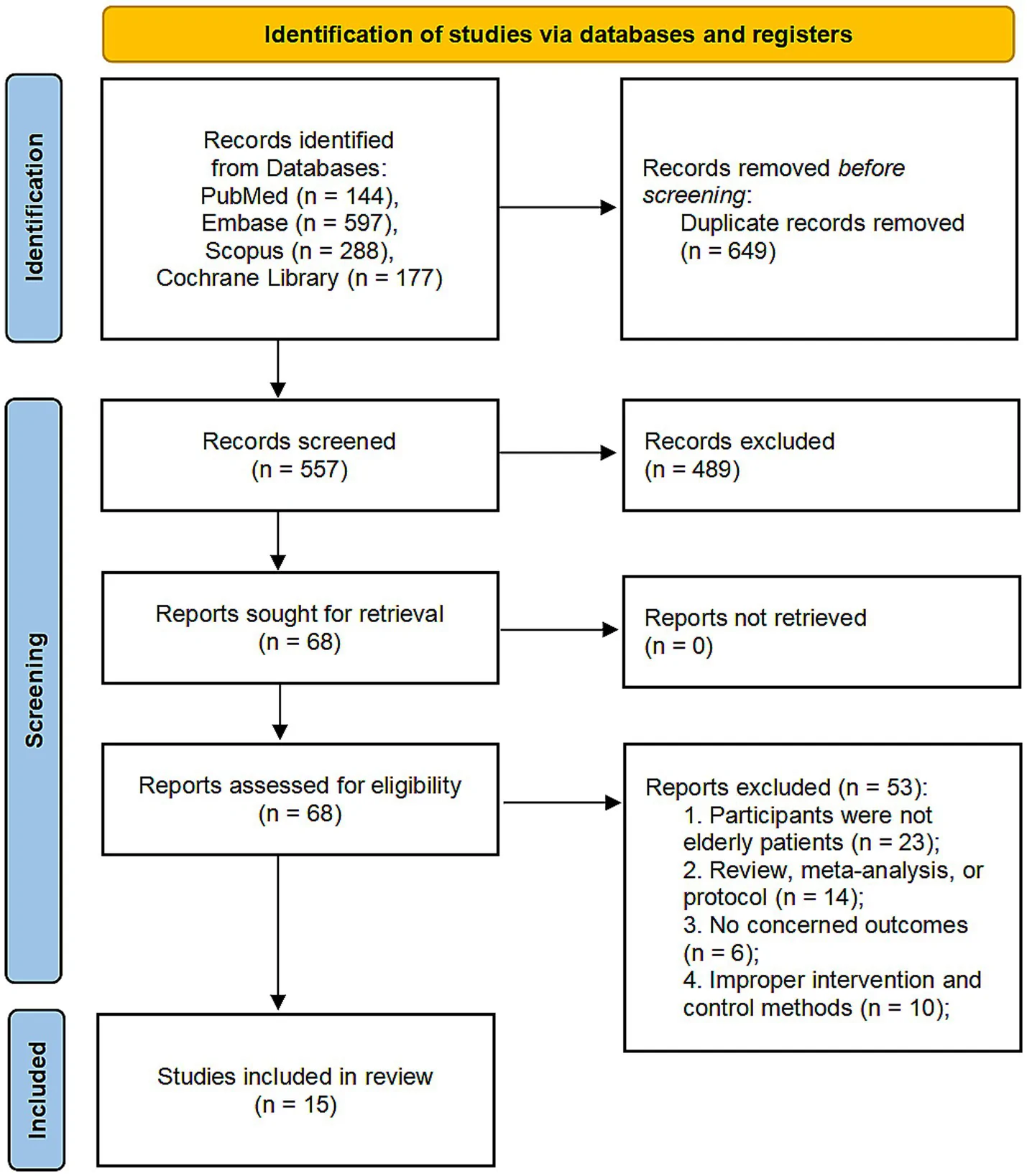
PRISMA 2020 flow diagram for this meta-analysis.

A total of 1,206 records were identified through the initial database search. After deduplication, 557 studies underwent title and abstract screening. Following this initial assessment, 68 articles were deemed eligible for full-text review. After detailed evaluation against the predefined inclusion and exclusion criteria, 15 RCTs (18–32) published between 2009 and 2026 were included, encompassing 3,274 elderly cardiac surgery patients (1,656 in the dexmedetomidine group and 1,618 in the control group). The detailed study selection process is illustrated in the PRISMA flow diagram (Figure 2).

The characteristics of the 15 included RCTs are presented in Table 1. Thirteen trials enrolled exclusively age-eligible older patients according to the thresholds used in this review, whereas Wang et al. (30) and Hao et al. (31) contributed extractable age-specific subgroup data for patients aged 65 years or older from the published report or Supplementary material. The included studies were clinically heterogeneous with respect to procedure type, timing and context of dexmedetomidine administration, and treatment duration. Surgical procedures included CABG, valve surgery, combined CABG-valve procedures, and minimally invasive off-pump CABG; dexmedetomidine was administered intraoperatively, postoperatively in the ICU, nocturnally, after extubation, or across the intraoperative-to-postoperative period. These differences are clinically important because surgical invasiveness, cardiopulmonary bypass exposure, sedative exposure, ICU trajectory, and baseline cardiovascular reserve may influence POD risk, delirium detection, length of stay, and bradycardia risk.

**Table 1:** Characteristics of included studies.

Study and design	Sample size (Intervention/control)	Population	Intervention and control methods	Length of Intervention	Assessment tools for delirium
Shehabi, 2009 (26), DB, MC	152/147	Patients ≥ 60 years old and undergoing pump cardiac surgery, including CABG, valve surgery, combination CABG, valve replacement procedures	DEX at 0.3 to 0.7 μg/kg/h; Morphine at 10 to 70 μg/kg/h	Started within 1 h of ICU admission, until discharge from ICU, total duration of study drug was 17.5 h	CAM-ICU
Djaiani, 2016 (20), SB, MC	91/92	Patients ≥ 60 years old undergoing elective complex cardiac surgery and ≥ 70 years old undergoing either isolated coronary revascularization or single-valve replacement surgery	DEX at 0.2 to 0.7 μg/kg/h; Propofol at 25 to 50 μg/kg/min	Started at ICU admission, lasted for a maximum of 24 h	CAM-ICU
Li 2017, (32), DB, MC	142/143	Patients ≥ 60 years old undergoing elective coronary artery bypass graft or valve replacement surgery	DEX at 0.1 to 0.6 μg/kg/h; Normal saline at the same rate	Started before surgery, until the end of mechanical ventilation, total duration of study drug was 18.7 h	CAM-ICU
Azeem, 2018 (18), DB, SC	30/30	Patients ≥ 60 years old undergoing elective cardiac surgery under general anesthesia	DEX at 0.4 to 0.7 μg/kg/h; Morphine at 10 to 50 μg/kg/h	Started at ICU admission, until the end of mechanical ventilation, for a maximum of 24 h	CAM-ICU
Shokri, 2019 (27), DB, SC	144/142	Patients ≥ 60 years old undergoing elective isolated CABG	DEX at 0.7 to 1.2 μg/kg/h; Clonidine at 1 to 2 μg/kg/h	Started at ICU admission, until discharge from ICU, for a maximum of 72 h	CAM-ICU
Subramaniam, 2019 (28), NB, SC	30/30*	Patients ≥ 60 years old undergoing CABG or mitral valve replacement requiring cardiopulmonary bypass	DEX at 0.1 to 1.4 μg/kg/h; Propofol at 20 to 100 μg/kg/min	Started at chest closure, continued for up to 6 h postoperatively or until extubation	CAM-ICU
Turan, 2020 (29), DB, MC	194/186	Patients ≥ 64 years old undergoing cardiac surgery with cardiopulmonary bypass,	DEX at 0.1 to 0.4 μg/kg/h; Normal saline at the same rate	Started before the surgical incision, lasted for 24 h	CAM-ICU
Dong, 2021 (21), DB, SC	132/141	Patients ≥ 65 years old undergoing elective cardiac surgery associated with a planned ICU stay of over 2 days	DEX at 0.2 to 1.2 μg/kg/h; Normal saline at the same rate	Started the day of surgery from 10 p.m. to 6 a.m. on the next day, for at least 3 nights	CAM-ICU
Gao, 2021 (22), NB, SC	20/20	Patients ≥ 65 years old undergoing elective minimally invasive off-pump CABG	DEX at 0.2 to 0.6 μg/kg/h; Normal saline at the same rate	Started before induction of anesthesia, until the end of the operation	DSM-IV
Momeni, 2021 (24), DB, SC	205/203	Patients ≥ 60 years old undergoing cardiac surgery with cardiopulmonary bypass	DEX at 0.4 μg/kg/h; Normal saline at the same rate	Started at chest closure, continued for 10 h postoperatively	CAM and CAM-ICU
Chitnis, 2022 (19), NB, SC	34/33	Patients ≥ 75 years undergoing CABG or aortic valve replacement surgery on cardiopulmonary bypass	DEX at 0.5 to 1.5 μg/kg/h; Propofol at 25 to 50 μg/kg/min	Started at chest closure, until extubation	ICDSC
Qu, 2022 (25), DB, SC	188/206	Patients ≥ 60 years undergoing a cardiac surgical procedure with planned postoperative admission to the cardiac surgical ICU for 24 h or more	DEX at 1 μg/kg over 40 min, maximal dose of 80 μg; Normal saline at the same rate	Started following extubation on the night of surgery and subsequently every night throughout the ICU stay for up 3 days	CAM
Wang, 2023 (30), DB, SC	36/99*	Age-specific subgroup of patients ≥ 65 years scheduled for open cardiac valve surgery with bypass	DEX at 0.4 μg/kg/h; Normal saline at the same rate	Started before induction of anesthesia, until the end of surgery	CAM-ICU
Huet, 2024 (23), DB, MC	165/166	Patient aged 65 years or older who underwent cardiac surgery with or without cardiopulmonary bypass	DEX at 0.1 to 1.4 μg/kg/h; Normal saline at the same rate	Started the day of surgery from 8 p.m. to 8 a.m. on the next day, until the discharge from ICU, for a maximum of 7 days, median duration of study drug was 3 days	CAM-ICU
Hao, 2026 (31), DB, MC	39/34*	Age-specific subgroup of patients ≥ 65 years undergoing elective cardiac surgery (valvuloplasty, CABG, or valvuloplasty combined CABG procedures).	DEX at 0.4 to 0.6 μg/kg/h; Normal saline at the same rate	Started over 10 min post-intubation, terminated 30 min prior to surgery end	CAM

ICU, Intensive Care Unit; DB, Double Blind; SB, Single Blind; NB, No Blind; MC, Multi-Center; SC, Single Center; CABG, Coronary Artery Bypass Grafting; CAM, Confusion Assessment Method; CAM-ICU, Confusion Assessment Method in Intensive Care Unit, ICDSC, Intensive Care Delirium Screening Checklist, DSM, Diagnostic and Statistical Manual of Mental Disorders; DEX, Dexmedetomidine.

*Sample sizes refer to the age-eligible or outcome-relevant comparison extracted for this review and may differ from the full randomized sample of the original trials. Wang, 2023 (30) and Hao, 2026 (31) contributed age-specific subgroup data extracted from the published article or Supplementary material. In Subramaniam, 2019 (28), only the dexmedetomidine-sedation versus propofol-sedation comparison was included from the factorial trial (30/30 patients), without double-counting shared arms.

Variation in delirium ascertainment was also clinically relevant. Across trials, POD was assessed using different diagnostic or screening instruments, including CAM, CAM-ICU, ICDSC, and DSM-based criteria; assessment frequency and follow-up windows differed; and some studies focused on ICU assessments whereas others extended assessment to the ward or in-hospital period. Assessor blinding was also not uniformly reported. These differences may influence the probability of detecting POD, particularly hypoactive delirium, and should be considered when interpreting the pooled incidence and duration estimates.

### Quality assessment

The methodological quality of the included studies was evaluated with RoB 2 (Figure 1). Overall risk of bias was judged as low in 9 studies, as some concerns in 5 studies, and as high in 1 study (22), mainly because of deviations from the intended interventions. These limitations were incorporated into the GRADE assessment and into the interpretation of sensitivity analyses. Outcome-specific RoB 2 judgments are summarized in Supplementary material 3.

Publication bias was assessed using funnel plots and Egger’s regression test (Supplementary material 3). These analyses were interpreted cautiously because most outcomes included fewer than 10 studies, limiting the power and reliability of small-study-effect tests. Potential asymmetry was noted for length of hospital stay and hypotension, and trim-and-fill analyses were exploratory rather than definitive. The results after trim-and-fill adjustment showed no substantial difference in the pooled effect estimates compared to the original analysis, suggesting that the presence of publication bias did not significantly alter the conclusions for these outcomes. For other outcomes, the funnel plots exhibited relative symmetry. This visual assessment was corroborated by Egger’s test. However, non-significant Egger’s tests were not interpreted as proof of absence of publication bias.

### Primary outcomes

**Figure 3:**
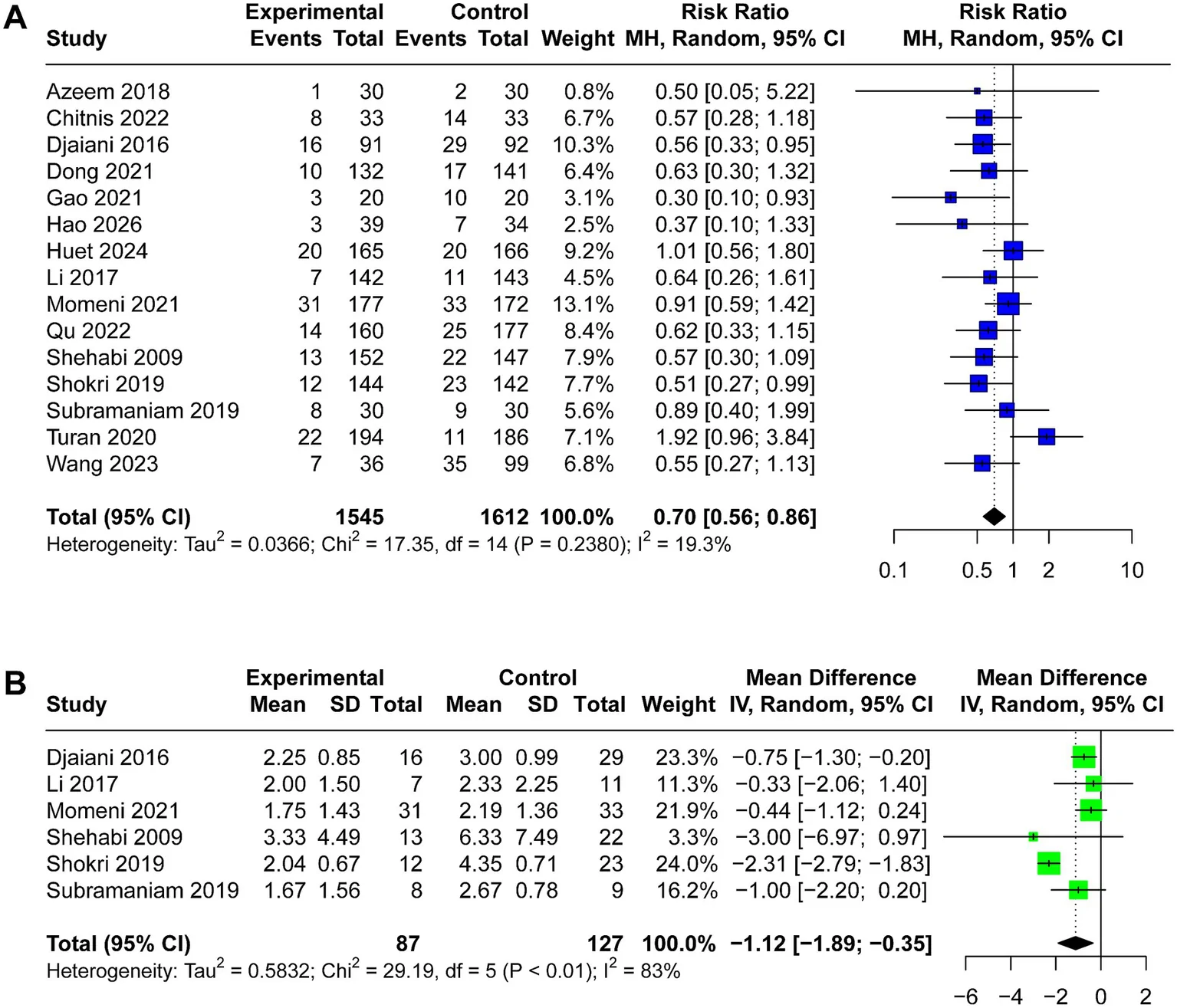
Forest plot comparing the effect of DEX versus comparative drugs on **(A)** incidence of POD, **(B)** duration of POD.

Fifteen trials including 3,157 analyzable participants contributed to the RR-based POD-incidence analysis. Perioperative dexmedetomidine was associated with a lower incidence of POD (RR 0.70, 95% CI 0.56 to 0.86, *p* = 0.001, I^2^ = 19%, Figure 3A; Table 2). This overall estimate should be interpreted as a broad synthesis across clinically different comparator contexts rather than as a single uniform treatment effect.

**Table 2:** Results of meta-analysis.

Outcome	Included studies, participants (DEX/control)	Effect (95% CI), *p* value, I^2^	*p* for subgroup difference
Incidence of POD			
Overall	15 studies, 1,545/1612	RR 0.70 (0.56 to 0.86), *p* = 0.001, I^2^ = 19%	
Comparator			
Saline/placebo	9 studies, 1,065/1138	RR 0.75 (0.55 to 1.03), *p* = 0.078, I^2^ = 41%	0.113
Sedative/analgesic	6 studies, 480/474	RR 0.59 (0.44 to 0.79), *p* = 0.0003, I^2^ = 0%	
Intervention duration			
≤ 24 h	11 studies, 944/986	RR 0.69 (0.52 to 0.92), *p* = 0.011, I^2^ = 32%	0.851
> 24 h	4 studies, 601/626	RR 0.69 (0.50 to 0.95), *p* = 0.023, I^2^ = 0%	
POD duration			
Overall	6 studies, 87/127	MD −1.12 (−1.89 to −0.35), *p* = 0.01, I^2^ = 83%	
Comparator			
Saline/placebo	2 studies, 38/44	MD −0.43 (−1.06 to 0.21), *p* = 0.191, I^2^ = 0%	0.001
Sedative/analgesic	4 studies, 49/83	MD −1.48 (−2.46 to −0.51), *p* = 0.008, I^2^ = 84%	
Intervention duration			
≤ 24 h	5 studies, 75/104	MD −0.68 (−1.07 to −0.28), *p* < 0.001, I^2^ = 0%	0.001
> 24 h	1 study, 12/23	MD −2.31 (−2.79 to −1.83), *P* < 0.001	
Length of ICU stay			
Overall	9 studies, 1,076/1087	MD −0.26 (−0.68 to 0.17), *p* = 0.21, I^2^ = 49%	
Comparator			
Saline/placebo	5 studies, 720/738	MD −0.29 (−0.62 to 0.04), *p* = 0.085, I^2^ = 0%	0.580
Sedative/analgesic	4 studies, 356/349	MD −0.34 (−1.30 to 0.61), *p* = 0.484, I^2^ = 78%	
Intervention duration			
≤ 24 h	6 studies, 579/573	MD −0.30 (−0.87 to 0.27), *p* = 0.299, I^2^ = 66%	0.404
> 24 h	3 studies, 497/514	MD −0.11 (−0.73 to 0.51), *p* = 0.731, I^2^ = 0%	
Length of hospital stay			
Overall	8 studies, 1,046/1057	MD −0.37 (−1.39 to 0.65), *p* = 0.51, I^2^ = 97%	
Comparator			
Saline/placebo	5 studies, 720/738	MD −0.24 (−1.63 to 1.16), *p* = 0.697, I^2^ = 95%	<0.001
Sedative/analgesic	3 studies, 326/319	MD −0.62 (−2.30 to 1.07), *p* = 0.51, I^2^ = 94%	
Intervention duration			
≤ 24 h	5 studies, 549/543	MD −0.43 (−1.75 to 0.88), *p* = 0.475, I^2^ = 90%	<0.001
> 24 h	3 studies, 497/514	MD −0.27 (−2.21 to 1.66), *p* = 0.826, I^2^ = 99%	
Bradycardia			
Overall	6 studies, 821/834	RR 2.08 (1.31 to 3.31), *p* = 0.0019, I^2^ = 0%	
Comparator			
Saline/placebo	3 studies, 495/515	RR 2.23 (0.81 to 6.15), *p* = 0.074, I^2^ = 4%	0.86
Sedative/analgesic	3 studies, 326/319	RR 2.49 (1.29 to 4.80), *p* = 0.007, I^2^ = 0%	
Intervention duration			
≤ 24 h	3 studies, 324/320	RR 1.98 (0.87 to 10.33), *p* = 0.005, I^2^ = 0%	0.55
> 24 h	3 studies, 497/514	RR 2.99 (0.87 to 10.33), *p* = 0.083, I^2^ = 0%	
Hypotension			
Overall	6 studies, 821/834	RR 1.09 (0.64 to 1.83), *p* = 0.76, I^2^ = 68%	
Comparator			
Saline/placebo	3 studies, 495/515	RR 1.55 (0.38 to 6.24), *p* = 0.588, I^2^ = 72%	0.63
Sedative/analgesic	3 studies, 326/319	RR 1.05 (0.48 to 2.25), *p* = 0.898, I^2^ = 76%	
Intervention duration			
≤ 24 h	3 studies, 324/320	RR 1.01 (0.49 to 2.06), *p* = 0.976, I^2^ = 76%	0.54
> 24 h	3 studies, 497/514	RR 1.65 (0.40 to 6.79), *p* = 0.498, I^2^ = 72%	

Six trials including 214 patients with POD contributed to delirium-duration analysis. Dexmedetomidine was associated with shorter duration of POD (MD −1.12 days, 95% CI −1.89 to −0.35 days, *p* = 0.01, I^2^ = 83%, Figure 3B; Table 2). Differences in delirium assessment frequency, ICU sedation protocols, comparator regimen, and definitions of delirium duration may have contributed to this inconsistency.

**Figure 4:**
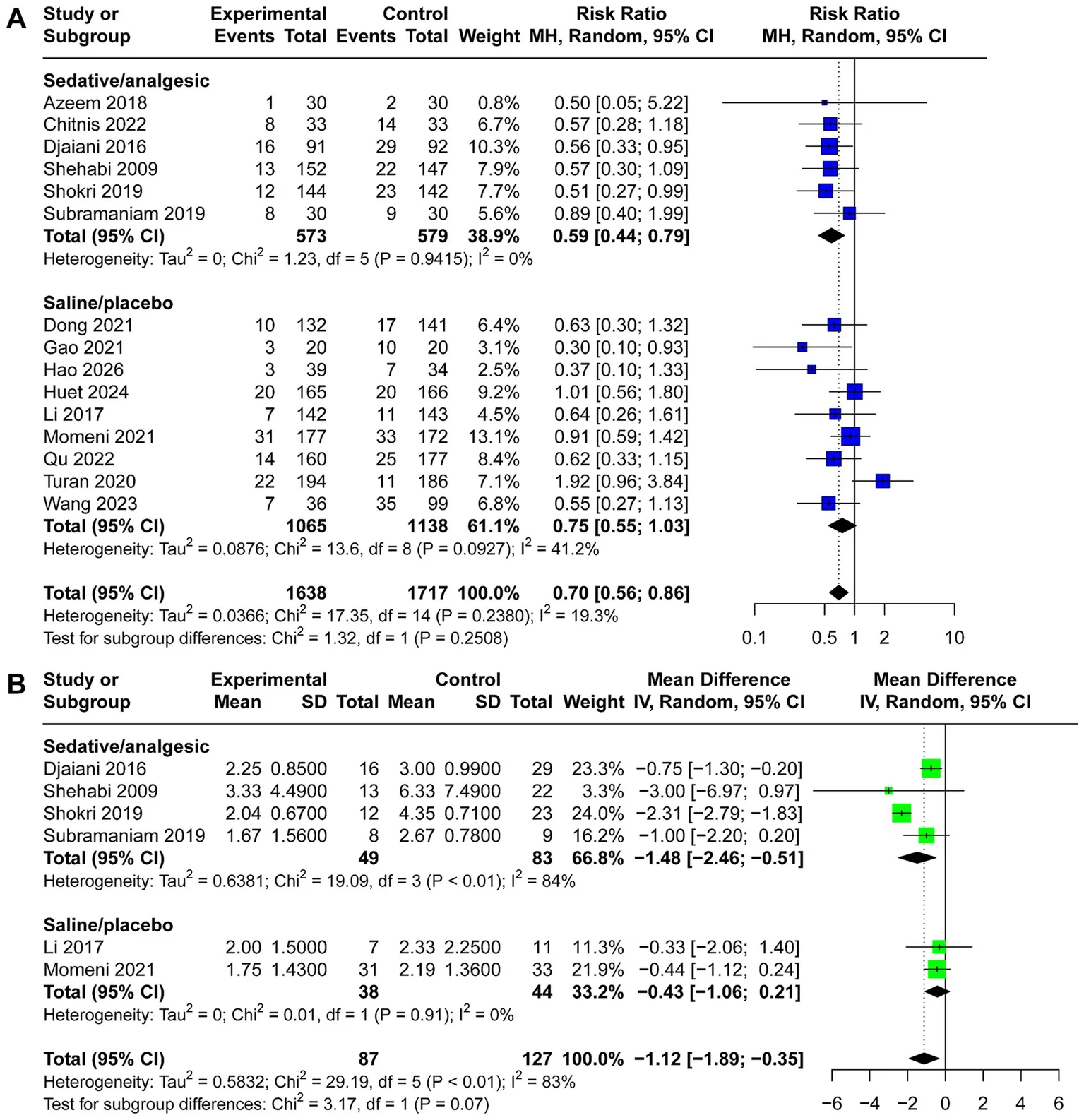
Subgroup analysis comparing sedative versus normal saline for **(A)** incidence of POD, **(B)** duration of POD.

In comparator-stratified analyses, the apparent reduction in POD was more evident in trials comparing dexmedetomidine with active sedative or analgesic regimens (RR 0.59, 95% CI 0.44 to 0.79, *p* = 0.0003, I^2^ = 0%, Figure 4A; Table 2) than in placebo/saline-controlled trials (RR 0.75, 95% CI 0.55 to 1.03, *p* = 0.078, I^2^ = 41%, Figure 4A; Table 2). A similar pattern was observed for delirium duration: the estimate was larger in active-comparator trials (MD −1.48 days, 95% CI −2.46 to −0.51 days, *p* = 0.0003, I^2^ = 84%, Figure 4B; Table 2) than in placebo/saline-controlled trials (MD −0.43 days, 95% CI −1.06 to 0.21 days, *p* = 0.91, I^2^ = 0%, Figure 4B; Table 2). This pattern suggests that part of the observed benefit may reflect replacement or avoidance of comparator regimens with different delirium-risk profiles, rather than an intrinsic neuroprotective effect of dexmedetomidine. For POD incidence, the subgroup difference by intervention duration was not statistically significant (*p* for subgroup difference = 0.851), and the comparator-based subgroup difference was also not statistically significant (*p* for subgroup difference = 0.113). Thus, the comparator pattern for POD incidence should be regarded as exploratory rather than definitive evidence of effect modification. For delirium duration, the comparator-based subgroup difference was statistically significant (*p* for subgroup difference = 0.001), but this analysis included few studies and substantial heterogeneity in the active-comparator subgroup.

### Secondary outcomes

**Figure 5:**
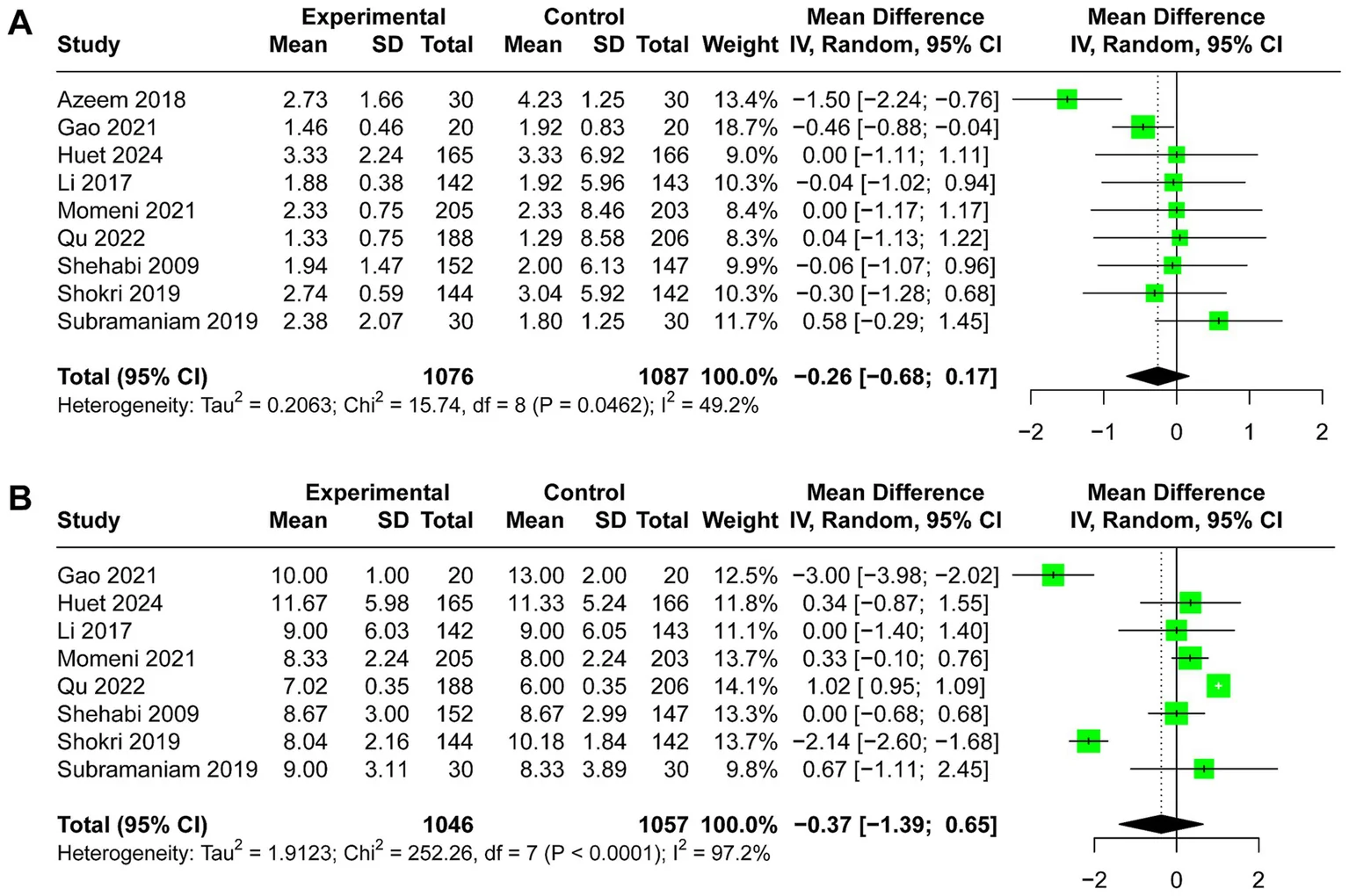
Forest plot comparing the effect of DEX versus comparative drugs on **(A)** length of ICU stay, **(B)** length of hospital stay.

Nine trials including 2,163 participants contributed to ICU length of stay. Dexmedetomidine was not associated with a statistically significant reduction in ICU stay (MD −0.26 days, 95% CI −0.68 to 0.17 days, *p* = 0.21, I^2^ = 49%, Figure 5A; Table 2). Eight trials including 2,103 participants contributed to hospital length of stay, with no statistically significant difference between groups (MD −0.37 days, 95% CI, −1.39 to 0.65 days, *p* = 0.51, I^2^ = 97%, Figure 5B; Table 2). The very high heterogeneity observed for length of hospital stay indicates substantial variability across studies, likely reflecting differences in surgical procedures, discharge criteria, postoperative care pathways, institutional practice, and baseline patient risk. Therefore, these findings do not support a consistent effect of dexmedetomidine on length of stay outcomes.

**Figure 6:**
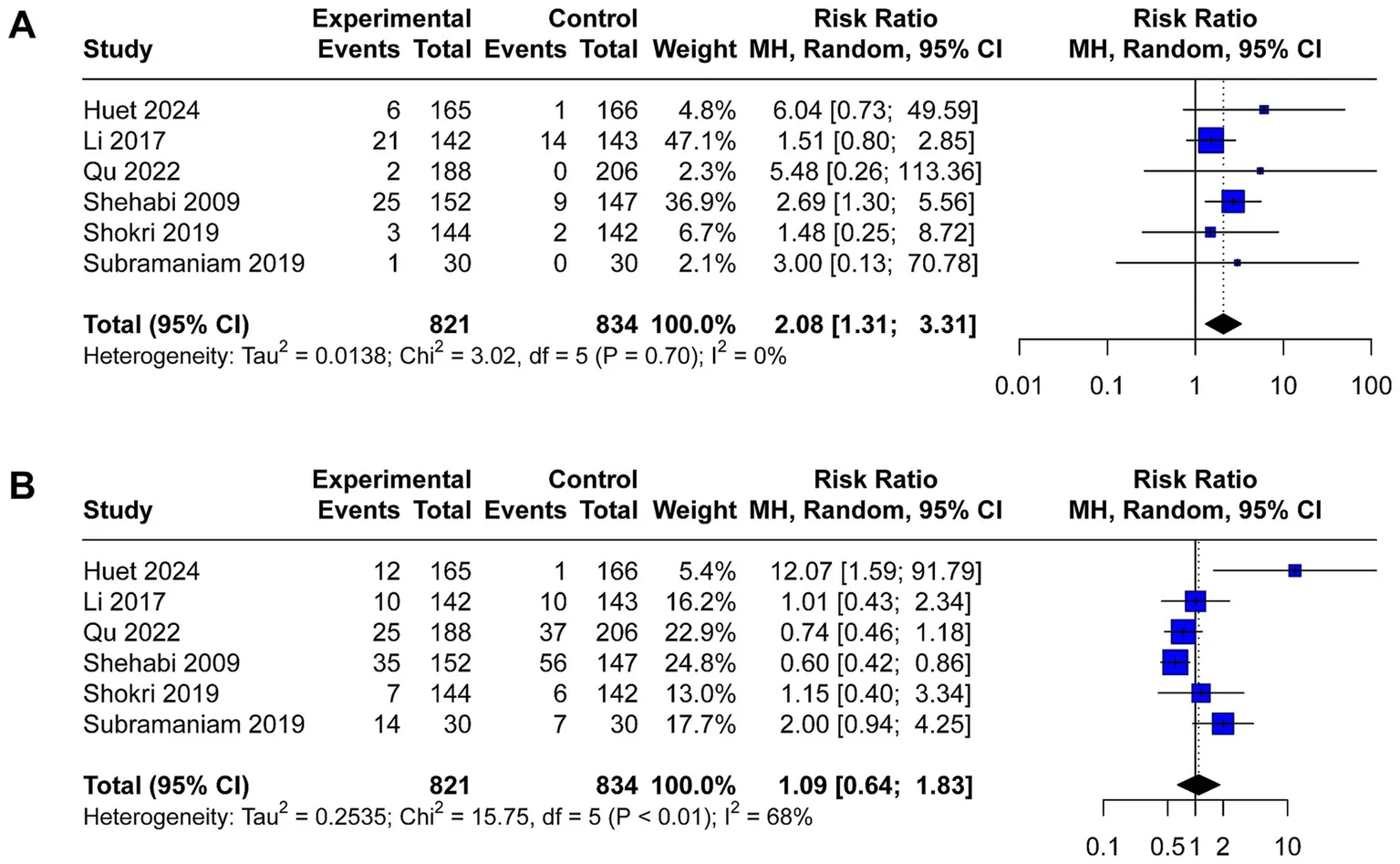
Forest plot comparing the effect of DEX versus comparative drugs on **(A)** bradycardia, **(B)** hypotension.

Six trials including 1,655 participants contributed to bradycardia and hypotension analyses. Dexmedetomidine was associated with an increased risk of bradycardia (RR 2.08, 95% CI 1.31 to 3.31, *p* = 0.0019, I^2^ = 0%, Figure 6A; Table 2). However, the clinical severity of bradycardia was inconsistently reported, and most trials did not clearly distinguish asymptomatic bradycardia from events requiring dose reduction, drug discontinuation, atropine, pacing, vasopressors, or other intervention. Dexmedetomidine was not associated with a statistically significant difference in hypotension (RR 1.09, 95%CI 0.64 to 1.83, *p* = 0.76, I^2^ = 68%, Figure 6B; Table 2), but definitions of hypotension varied across trials.

### Sensitivity and subgroup analyses

A leave-one-out sensitivity analysis was performed for all outcomes (Supplementary material 3). Because Gao et al. (22) was judged to be at high risk of bias and showed a large point estimate for POD incidence, we explicitly examined the pooled effect after excluding this study. The association between dexmedetomidine and lower POD incidence remained statistically significant after excluding Gao et al. (22) (RR 0.72, 95% CI 0.59 to 0.88), indicating that the primary POD finding was not driven solely by this trial. Similarly, the lack of significant effect on ICU length of stay, hospital length of stay, and hypotension was not altered in leave-one-out analyses. Furthermore, since excluding the factorial Subramaniam et al. (28) yielded similar estimates for POD incidence, delirium duration, bradycardia, and hypotension; the ICU length-of-stay estimate changed directionally and was therefore treated as exploratory. For length of stay outcomes, excluding studies in which medians/IQRs were converted to means/SDs did not show a statistically significant reduction in ICU stay (MD −0.39 days, 95% CI −1.02 to 0.25) or hospital stay (MD −1.22 days, 95% CI −2.71 to 0.28). The Hartung-Knapp-Sidik-Jonkman adjustment sensitivity analyses were consistent with the primary analyses (Supplementary material 3). Overall, these findings support the robustness of the primary results, while indicating appropriately wider uncertainty estimates for some outcomes.

Predefined subgroup analyses were performed by intervention duration (Supplementary material 3). For the primary outcomes, subgroup estimates by intervention duration were broadly consistent. For bradycardia, the direction of effect was also consistent across duration subgroups, although subgroup findings should be interpreted cautiously because few studies contributed to each comparison.

### GRADE assessment

The GRADE certainty of evidence is summarized in Supplementary material 4. Certainty was rated as moderate for POD incidence, mainly because of indirectness related to comparator and clinical heterogeneity. Certainty was low for delirium duration because only six trials contributed data and heterogeneity was substantial, leading to downgrading for inconsistency and imprecision. Certainty was low for ICU length of stay and very low for hospital length of stay; the latter was downgraded for very serious inconsistency and imprecision. Certainty was low for bradycardia and hypotension because of indirectness, incomplete reporting of adverse-event severity, inconsistent definitions of clinically significant bradycardia and hypotension, inconsistency, and imprecision. These ratings indicate that the pooled estimates for delirium duration and hospital length of stay are not clinically robust unless interpreted alongside their substantial heterogeneity, limited study numbers, and incompletely explained sources of between-trial variation.

The definition and ascertainment of delirium duration differed across contributing trials. Djaiani et al. (20) recorded onset and duration among patients with delirium and reported duration in days. Li et al. (32) reported duration among patients who developed delirium during the first five postoperative days. Momeni et al. (24) reported duration among patients with in-hospital POD, with ICU assessments using CAM-ICU and ward assessments using CAM. Shehabi et al. (26) reported delirium days during the early postoperative follow-up period. Shokri et al. (27) followed delirious patients until 8 days after surgery and considered patients delirium-free when CAM-negative for more than 24 h. Subramaniam et al. (28) reported in-hospital delirium duration using CAM/CAM-ICU assessments. All values were harmonized to days for pooling, but differences in assessment frequency, follow-up window, and criteria for delirium resolution likely contributed to the high heterogeneity.

## Discussion

This updated, age-specific systematic review and meta-analysis of 15 RCTs involving 3,274 patients suggests that perioperative dexmedetomidine may reduce POD incidence in selected elderly patients undergoing cardiac surgery. However, the strength and generalizability of this conclusion are limited by moderate certainty of evidence, comparator heterogeneity, risk-of-bias concerns, and substantial clinical heterogeneity. The delirium-duration finding should be interpreted as an average association across heterogeneous studies rather than as evidence of a uniform treatment effect, because heterogeneity was high and certainty was low. For clinical interpretation, the pooled control event rate for POD was 16.6% in the RR-based analyzable population. Applying the pooled RR of 0.70 corresponds to an absolute risk reduction of approximately 5.0%, or about 20 patients treated to prevent one POD event. For bradycardia, the pooled control event rate was 3.1%; applying the pooled RR of 2.08 corresponds to an absolute risk increase of approximately 3.4%, or about 30 patients treated for one additional reported bradycardia event. These absolute estimates should be interpreted cautiously because baseline risk varied across trials.

Comparator type is central to interpreting the pooled effect. For POD incidence, the saline/placebo subgroup did not show a statistically significant reduction, and the formal test for subgroup difference by comparator type was not statistically significant (*p* = 0.113). Thus, the data do not establish a comparator-by-treatment interaction or prove a neuroprotective mechanism of dexmedetomidine. The apparently larger effect in active-comparator trials may partly reflect avoidance of sedative or analgesic strategies that can increase delirium risk in some contexts, rather than a specific mechanistic benefit of dexmedetomidine itself. Therefore, the most cautious interpretation is that dexmedetomidine may be a reasonable alternative to selected sedative regimens in selected elderly cardiac surgery patients, while its incremental benefit over saline/placebo or usual care remains less certain. Dexmedetomidine was also associated with a consistent increase in reported bradycardia, warranting careful hemodynamic monitoring in this elderly population.

### Comparison to previous literature

Our findings are broadly consistent with previous meta-analyses examining dexmedetomidine for delirium prevention in ICU and surgical populations (6, 8, 33–35). Lewis et al. (6) included 77 RCTs of mechanically ventilated adult ICU patients and reported that dexmedetomidine reduced delirium risk and modestly decreased the duration of mechanical ventilation and ICU stay, while increasing of bradycardia risk. Poon et al. (7), focusing on adult cardiac surgery patients, reported lower POD incidence, shorter ICU stay and mechanical ventilation duration, and lower short-term mortality. Most recently, Caetano da Silva et al. (8) analyzed 31 RCTs in cardiac surgery patients and found lower POD incidence with dexmedetomidine, an increased bradycardia risk, and no significant effects on mortality or length of hospital stay.

These prior reviews provide important context, but the present analysis contributes additional evidence in several ways. First, it focuses specifically on elderly cardiac surgery patients, who have higher baseline POD risk and may be more vulnerable to sedative-related hemodynamic adverse events. Second, it emphasizes comparator-dependent interpretation by distinguishing dexmedetomidine versus active sedative regimens from dexmedetomidine versus saline or placebo. This distinction is important for determining whether dexmedetomidine may be preferable to selected alternative sedation strategies rather than uniformly effective across comparator contexts. Third, the review gives greater attention to geriatric safety and clinical interpretability, including the bradycardia signal and the limited reporting of clinically significant bradycardia.

The increased risk of bradycardia observed in this review is consistent with prior meta-analyses and is clinically relevant for older patients, who may be more susceptible to hemodynamic instability. Unlike findings from broader ICU populations (6), we did not observe significant reductions in ICU or hospital length of stay. This may reflect the postoperative course of older cardiac surgery patients, in whom recovery is influenced by frailty, surgical complexity, complications, rehabilitation needs, and institutional discharge practices (36).

The secondary outcomes therefore require a more cautious interpretation than the POD-incidence outcome. Although delirium duration was statistically shorter in the pooled analysis, the high heterogeneity suggests that the estimate may reflect differences in delirium assessment frequency, criteria for delirium resolution, follow-up duration, comparator regimens, ICU sedation protocols, and baseline delirium risk rather than a uniform treatment effect. Similarly, the hospital length-of-stay analysis had very high heterogeneity and no statistically significant pooled reduction, limiting its usefulness for inferring a predictable reduction in resource use.

In summary, this review provides an age-focused synthesis of randomized evidence, but its clinical implications should remain measured. The findings support considering dexmedetomidine as one possible component of individualized sedation strategies in selected elderly cardiac surgery patients, while recognizing uncertainty related to comparator type, high heterogeneity for delirium duration and hospital length of stay, and increased bradycardia risk. Clinical heterogeneity is central to this interpretation: CABG, valve surgery, and off-pump surgery differ in procedural invasiveness, cardiopulmonary bypass exposure, anesthetic approach, ICU course, baseline delirium risk, sedative exposure, and postoperative recovery trajectory. Similarly, intraoperative, postoperative, nocturnal, ICU-based, and post-extubation dexmedetomidine protocols may represent different clinical interventions rather than a single uniform exposure.

Importantly, the pooled POD estimate should not be interpreted as fully independent of delirium-surveillance intensity. Trials with more frequent assessments, blinded assessors, ICU-plus-ward surveillance, or longer follow-up may detect more events than trials with less intensive screening. Because hypoactive delirium can be missed without structured and repeated assessment, between-trial differences in ascertainment may have contributed to residual heterogeneity and potential underdetection.

### Implications for practice and research

The observed reduction in POD incidence with dexmedetomidine may be clinically relevant because delirium affects 20 to 50% of elderly cardiac surgery patients and is associated with increased morbidity, mortality, prolonged hospitalization, and long-term cognitive decline (37). As a highly selective α2-adrenoceptor agonist, dexmedetomidine provides sedation with limited respiratory depression and may produce sedation patterns that differ from those of some GABAergic sedatives (8). Experimental and translational studies have also suggested potential effects on inflammatory signaling, microglial activation, and blood–brain barrier integrity (38, 39). However, sleep architecture, neuroinflammatory modulation, microglial activity, and blood–brain barrier function were not measured as outcomes in the included trials. These pathways should therefore be considered biologically plausible, hypothesis-generating explanations rather than mechanisms demonstrated by this meta-analysis.

The comparator-dependent findings should also be interpreted with nuance. Benzodiazepines are well recognized as delirium-promoting in older adults, whereas the effects of propofol and opioids may depend on dose, depth of sedation, analgesic strategy, duration of exposure, mechanical ventilation status, and ICU protocols. Therefore, the apparent benefit of dexmedetomidine versus active comparators may reflect differences among specific sedation strategies rather than a simple contrast between dexmedetomidine and uniformly deliriogenic alternatives. The significant reduction in delirium duration observed in our analysis should likewise be interpreted cautiously because heterogeneity was substantial, and the pooled estimate should be viewed as an average effect across diverse perioperative contexts.

The increased incidence of bradycardia associated with dexmedetomidine is consistent with its known pharmacological effects on cardiac conduction through central and peripheral alpha-2-adrenoceptor stimulation (40). This safety signal is clinically important in older cardiac surgery patients, who may have pre-existing conduction disease, beta-blocker or other negative chronotropic medication use, reduced physiologic reserve, and perioperative hemodynamic instability. This finding necessitates careful patient selection and monitoring. Elderly cardiac surgery patients often have pre-existing conduction abnormalities, reduced cardiac reserve, and may be taking negative chronotropic medications, factors that could predispose them to symptomatic bradycardia (41). However, the pooled estimate primarily reflects reported event counts, and most included trials did not consistently distinguish transient asymptomatic bradycardia from clinically significant episodes requiring dose reduction or interruption, atropine, vasopressors, temporary pacing, or other intervention. Thus, while dexmedetomidine appears to increase reported bradycardia, the clinical severity and downstream consequences of these events remain incompletely characterized. Clinicians should exercise caution when administering dexmedetomidine to patients with pre-existing bradycardia, advanced heart block, severe left ventricular dysfunction, or limited hemodynamic reserve, and continuous cardiac monitoring with readiness to adjust or interrupt infusion rates remains essential (42). The apparent difference in bradycardia risk between comparator subgroups may reflect differences in baseline hemodynamic risk, co-administered medications, or sedation protocols, and should not be interpreted as definitive evidence of comparator-specific safety modification.

Safety findings should be interpreted clinically rather than as event counts alone. Although hypotension was not significantly increased in the pooled analysis, hemodynamic instability remains a major concern in older cardiac surgical patients (43), and the available trial reports did not consistently distinguish transient blood-pressure changes from events requiring treatment escalation, dose interruption, vasopressors, or other rescue interventions.

The hypotension finding also requires caution. Although the pooled analysis did not show a statistically significant increase in hypotension, trial definitions varied, including different blood-pressure thresholds, relative declines from baseline, or treatment-triggered definitions. Reporting of vasopressor use, fluid treatment, dose adjustment, or clinical sequelae was incomplete, limiting interpretation of this safety endpoint.

Although dexmedetomidine was associated with lower POD incidence, the delirium-duration estimate was based on only 214 affected patients and showed substantial heterogeneity, and neither ICU nor hospital length of stay was significantly reduced in the main analysis. Length of stay is a multifactorial outcome influenced by surgical complexity, postoperative complications, institutional discharge practices, rehabilitation needs, and availability of step-down or post-acute care (44, 45). The considerable heterogeneity in hospital length of stay further limits confidence in this pooled estimate. Accordingly, the clinical implications of dexmedetomidine should be framed primarily around a possible reduction in POD incidence and the need for hemodynamic safety monitoring, rather than broader claims of shortened delirium duration, improved postoperative recovery, or reduced resource utilization.

## Limitations

This meta-analysis has several limitations that should temper interpretation of the findings. First, the review was restricted to English-language publications, which may have introduced language bias. In addition, although multiple databases were searched, potential limitations in search strategy sensitivity and indexing cannot be fully excluded.

Second, substantial clinical heterogeneity was present across the included trials. Studies differed in surgery type, including CABG, valve surgery, and off-pump surgery; timing of dexmedetomidine use, including intraoperative, postoperative, nocturnal, ICU-based, and post-extubation administration; dose range; and treatment duration, ranging from hours to days. These differences may affect delirium risk, sedative exposure, ICU course, discharge practices, and bradycardia risk, and they cannot be fully resolved by random-effects models or subgroup analyses. Comparator regimens were also heterogeneous, including saline or placebo as well as different active sedative or analgesic drugs, which limits the interpretation of a single pooled effect.

Third, risk-of-bias concerns should temper interpretation of the results. Five trials were judged as having some concerns and one trial, Gao et al. (22), was judged as high risk of bias. Although the POD incidence finding remained statistically significant after excluding Gao et al. (22), these judgments indicate that the pooled estimates should not be interpreted as high-certainty evidence.

Fourth, the handling of multi-arm trials may influence pooled estimates if shared control groups are not addressed consistently; therefore, this issue should be considered when interpreting comparator-specific subgroup findings.

Fifth, the formal GRADE assessment showed that certainty was not high for any pooled outcome and was low or very low for several clinically important outcomes, particularly delirium duration and hospital length of stay, because of serious inconsistency, imprecision, and indirectness.

Sixth, although all included trials used validated delirium screening tools (such as the CAM-ICU), variations in screening frequency, assessment timing, and follow-up duration may have led to underdetection of hypoactive delirium, which is highly prevalent but frequently missed in older adults. Moreover, limited information was available regarding delirium severity, which restricted our ability to determine whether dexmedetomidine influenced the clinical intensity or burden of delirium beyond event occurrence and duration.

Finally, the included trials lacked long-term follow-up data (e.g., 6 months to 1 year postoperatively), precluding an assessment of whether dexmedetomidine-associated reduction in POD translates into improved long-term cognitive function, quality of life, or mortality. Additional limitations relate to data-source transparency and complex study designs: several included sample sizes represent age-eligible subgroups rather than the full trial populations, and Subramaniam et al. (28) involved a factorial design that may introduce co-intervention complexity. These issues were explored in sensitivity analyses but cannot be fully resolved without individual-patient data.

Safety outcomes were also limited by incomplete harmonization and reporting. Bradycardia was usually pooled as a binary event, but trial reports often did not clarify whether events were mild and asymptomatic or required active treatment, dose reduction, discontinuation, atropine, vasopressors, pacing, or other intervention. Similarly, hypotension definitions differed across trials and were not consistently linked to treatment intensity or clinical consequences. These limitations reduce the clinical interpretability of the pooled safety estimates.

An additional limitation is variation in delirium assessment across trials. Diagnostic tools, assessment frequency, assessor blinding, assessment setting (ICU only versus ICU plus ward), and follow-up duration were not uniform. This variation may have affected POD detection and delirium-duration estimates, especially for hypoactive delirium, and could not be fully incorporated into the quantitative synthesis because trial-level reporting was incomplete.

Future large-scale, multicenter RCTs should prespecify comparator strategy, use standardized delirium assessment schedules across ICU and ward settings, report delirium severity and duration consistently, and distinguish clinically significant bradycardia from asymptomatic events. Future trials should also evaluate long-term cognitive and functional outcomes, include frailty and baseline cognitive assessments, and report dosing, loading, discontinuation, and rescue-intervention protocols in sufficient detail to guide individualized perioperative sedation decisions.

## Conclusion

This updated systematic review and meta-analysis suggests that perioperative dexmedetomidine may reduce POD in selected elderly patients undergoing cardiac surgery, with moderate-certainty evidence for POD incidence. The apparent benefit is more evident when dexmedetomidine is compared with active sedative or analgesic regimens than with placebo/saline, and this distinction should guide interpretation. Evidence for shorter delirium duration was low certainty, based on few affected patients, and highly heterogeneous; therefore, it should be interpreted as exploratory and should not drive the main conclusion. Dexmedetomidine increases bradycardia, but the clinical severity of this adverse event was incompletely reported. Overall, dexmedetomidine should be considered as a potential component of individualized delirium-prevention strategies rather than as a definitive or universally protective intervention.

## Data Availability

The original contributions presented in the study are included in the article/Supplementary material, further inquiries can be directed to the corresponding author.

## Footnotes

1
https://osf.io/f6nwu
