# Supplementary material for: Efficacy and hemodynamic safety of perioperative dexmedetomidine for the prevention of postoperative delirium in elderly patients undergoing cardiac surgery: an updated systematic review and meta-analysis of randomized controlled trials
Source: Front Neurol. 2026 Aug 12;17:1895312. doi: 10.3389/fneur.2026.1895312 (PMC13506365; doi:10.3389/fneur.2026.1895312)
Supplement: Supplementary file 2 [file Supplementary_file_2.DOCX]

**Supplementary Material 2: Protocol registration and revision history, search strategy in PubMed, Embase, Scopus and Cochrane library**

The review protocol was registered on the Open Science Framework on Oct 21, 2025 (https://osf.io/f6nwu). No major deviations from the review question, population, intervention, comparator framework, or primary outcomes were made. The following clarifications and post hoc additions were made during revision in response to reviewer and editor comments.

| **Item** | **Timing** | **Description** | **Rationale/impact** |
| --- | --- | --- | --- |
| Original protocol registration | Oct 21, 2025 | Protocol registered on the Open Science Framework (https://osf.io/f6nwu). | Not applicable. |
| Database search update | Revision stage | Searches were updated through July 12, 2026, and database-specific strategies were reported in Supplementary Material 2. | This was an update of the search date and did not change the review question. |
| Eligibility clarification | Revision stage | The eligibility criteria were clarified to specify inclusion of trials in which all randomized participants were aged 60 years or older or trials with extractable age-specific data for patients aged 60 years or older. | This clarified the age-specific scope of the review and the handling of Wang 2023 and Hao 2026. |
| Handling of factorial trial | Revision stage | For Subramaniam 2019, only the dexmedetomidine-sedation versus propofol-sedation comparison relevant to the review question was extracted, and shared arms were not double counted. | This clarified the handling of the factorial design and was explored in sensitivity analysis. |
| Sensitivity analyses | Revision stage | Additional sensitivity analyses were performed excluding Gao 2021, excluding Subramaniam 2019, and excluding studies in which medians/IQRs were converted to means/SDs for length-of-stay outcomes. | These analyses were added in response to reviewer/editor requests and did not change the primary review question. |
| Hartung-Knapp-Sidik-Jonkman analysis | Revision stage | HKSJ random-effects sensitivity analyses were added to assess uncertainty in random-effects estimates, particularly for outcomes with few studies or heterogeneity. | This strengthened statistical robustness and was added after peer review. |
| Subgroup-interaction testing | Revision stage | Formal tests for subgroup differences were reported for comparator type and intervention duration where data were available. | This was added to avoid interpreting subgroup significance patterns as evidence of effect modification without interaction testing. |
| Absolute-effect estimates | Revision stage | Absolute effects were added for POD incidence and bradycardia, including approximate absolute risk reduction/increase and number-needed-to-treat/harm. | This was added to improve clinical interpretability. |
| GRADE assessment | Revision stage | A GRADE certainty-of-evidence assessment was added for each pooled outcome. | This was added in response to reviewer comments and was used to moderate the interpretation of the conclusions. |
| Outcome-specific RoB 2 reporting | Revision stage | RoB 2 judgments were clarified as outcome-level assessments, with outcome-specific judgments summarized in supplementary materials. | This was added in response to the editor's request to state explicitly whether RoB 2 was performed separately for each outcome. |

Overall, these changes were made to improve transparency, reproducibility, and clinical interpretability. They did not alter the primary review question but led to more cautious interpretation of the pooled estimates.

Searching strategies

| Database  (number of reports) | Key word terms |
| --- | --- |
| Pubmed  N=144 | (("Thoracic Surgery"[Mesh] OR "Cardiac Surgical Procedures"[Mesh] OR cardiac surger*[tiab] OR heart surger*[tiab] OR coronary bypass[tiab] OR CABG[tiab] OR valve surger*[tiab] OR valvular surger*[tiab] OR aortic surger*[tiab] OR TAVR[tiab] OR transcatheter aortic valve[tiab]) AND ("Dexmedetomidine"[Mesh] OR dexmedetomidine[tiab] OR Precedex[tiab] OR Dexdor[tiab]) AND ("Delirium"[Mesh] OR delirium[tiab] OR "postoperative delirium"[tiab] OR "post-operative delirium"[tiab] OR "cognitive dysfunction"[tiab] OR "postoperative cognitive dysfunction"[tiab] OR "postoperative neurocognitive disorder"[tiab] OR "post-operative neurocognitive disorder"[tiab]) AND (randomized controlled trial[pt] OR controlled clinical trial[pt] OR random*[tiab] OR placebo[tiab] OR trial[tiab])) |
| Embase  N=597 | ('heart surgery'/exp OR 'thoracic surgery'/exp OR 'cardiac surgical procedure'/exp OR 'coronary artery bypass grafting'/exp OR 'heart valve surgery'/exp OR cardiac surger*:ti,ab OR heart surger*:ti,ab OR CABG:ti,ab OR 'coronary bypass':ti,ab OR 'valve surgery':ti,ab OR 'valvular surgery':ti,ab OR TAVR:ti,ab OR 'transcatheter aortic valve':ti,ab) AND ('dexmedetomidine'/exp OR dexmedetomidine:ti,ab OR precedex:ti,ab OR dexdor:ti,ab) AND ('delirium'/exp OR delirium:ti,ab OR 'postoperative delirium':ti,ab OR 'post operative delirium':ti,ab OR 'cognitive dysfunction':ti,ab OR 'postoperative cognitive dysfunction':ti,ab OR 'postoperative neurocognitive disorder':ti,ab) AND ('randomized controlled trial'/exp OR random*:ti,ab OR placebo:ti,ab OR trial:ti,ab) |
| Scopus  N=288 | TITLE-ABS-KEY(("cardiac surgery" OR "heart surgery" OR "thoracic surgery" OR "coronary bypass" OR CABG OR "valve surgery" OR "valvular surgery" OR "aortic surgery" OR TAVR OR "transcatheter aortic valve") AND (dexmedetomidine OR Precedex OR Dexdor) AND (delirium OR "postoperative delirium" OR "post-operative delirium" OR "cognitive dysfunction" OR "postoperative cognitive dysfunction" OR "postoperative neurocognitive disorder" OR "post-operative neurocognitive disorder") AND (random* OR placebo OR trial)) |
| Cochrane  N=177 | #1 MeSH descriptor: [Cardiac Surgical Procedures] explode all trees  #2 MeSH descriptor: [Thoracic Surgery] explode all trees  #3 ("cardiac surgery" OR "heart surgery" OR "coronary bypass" OR CABG OR "valve surgery" OR "valvular surgery" OR "aortic surgery" OR TAVR OR "transcatheter aortic valve"):ti,ab,kw  #4 #1 OR #2 OR #3  #5 MeSH descriptor: [Dexmedetomidine] explode all trees  #6 (dexmedetomidine OR Precedex OR Dexdor):ti,ab,kw  #7 #5 OR #6  #8 MeSH descriptor: [Delirium] explode all trees  #9 (delirium OR "postoperative delirium" OR "post-operative delirium" OR POD OR "cognitive dysfunction" OR "postoperative cognitive dysfunction" OR "post-operative cognitive dysfunction" OR "postoperative neurocognitive disorder" OR "post-operative neurocognitive disorder"):ti,ab,kw  #10 #8 OR #9  #11 (random* OR trial OR placebo OR "controlled trial"):ti,ab,kw  #12 #4 AND #7 AND #10 AND #11 |
