# Supplementary material for: Efficacy and hemodynamic safety of perioperative dexmedetomidine for the prevention of postoperative delirium in elderly patients undergoing cardiac surgery: an updated systematic review and meta-analysis of randomized controlled trials
Source: Front Neurol. 2026 Aug 12;17:1895312. doi: 10.3389/fneur.2026.1895312 (PMC13506365; doi:10.3389/fneur.2026.1895312)
Supplement: Supplementary file 3 [file Supplementary_file_3.DOCX]

**Supplementary Material 3:** Publication bias assessment by funnel plot and Egger’s test, forest plot after trim-and-fill adjustment, sensitivity and subgroup analyses, outcome-specific RoB 2 assessment.

Figure 1: Funnel plot for (A) incidence of delirium, (B) duration of delirium, (C) length of ICU stay, (D) length of hospital stay, (E) incidence of hypotension, (F) incidence of bradycardia

Figure 2: Forest plot after trim-and-fill method for (A) length of hospital stay, (B) incidence of hypotension

Figure 3: Sensitivity analysis for (A) incidence of delirium, (B) duration of delirium, (C) length of ICU stay

Figure 4: Sensitivity analysis for (A) length of hospital stay, (B) incidence of bradycardia, (C) incidence of hypotension

Figure 5: Subgroup analysis comparing length of intervention ≤ 24 hours versus > 24 hours for (A) incidence of POD, (B) duration of POD

Figure 6: Subgroup analysis for length of ICU stay, (A) length of intervention ≤ 24 hours versus > 24 hours, (B) sedative versus normal saline

Figure 7: Subgroup analysis for length of hospital stay, (A) length of intervention ≤ 24 hours versus > 24 hours, (B) sedative versus normal saline

Figure 8: Subgroup analysis for incidence of bradycardia, (A) sedative versus normal saline, (B) length of intervention ≤ 24 hours versus > 24 hours

Figure 9: Subgroup analysis for incidence of hypotension, (A) sedative versus normal saline, (B) length of intervention ≤ 24 hours versus > 24 hours

Table 1: Outcome-specific RoB 2 assessment

| **Outcome** | **Contributing studies** | **Low risk** | **Main reasons for concerns** |
| --- | --- | --- | --- |
| POD incidence | 15 RCTs | Low risk: 9;  Some concerns: 5; High risk: 1 | Open-label or single-blind designs in some trials, missing delirium-assessment data in age-eligible subgroups, and high risk in Gao 2021 related mainly to deviations from intended interventions. |
| Delirium duration | 6 RCTs | Low risk: 4;  Some concerns: 2; High risk: 0 | Some concerns mainly reflected assessor blinding and incomplete reporting of how duration was ascertained across delirium-positive participants. |
| ICU length of stay | 9 RCTs | Low risk: 6;  Some concerns: 2; High risk: 1 | High risk in Gao 2021 and some concerns in trials with open-label or complex co-intervention structures; length-of-stay outcomes were also vulnerable to non-normal distributions and conversion of medians/IQRs. |
| Hospital length of stay | 8 RCTs | Low risk: 5;  Some concerns: 2; High risk: 1 | High risk in Gao 2021, some concerns in open-label or complex co-intervention studies, and vulnerability to skewed discharge-time data. |
| Bradycardia | 6 RCTs | Low risk: 4;  Some concerns: 2; High risk: 0 | Some concerns mainly reflected inconsistent reporting of bradycardia definitions, severity, and treatment requirements rather than event ascertainment alone. |
| Hypotension | 6 RCTs | Low risk: 4;  Some concerns: 2; High risk: 0 | Some concerns mainly reflected inconsistent definitions of hypotension and incomplete reporting of clinical management or treatment intensity. |

Table 2: Exclusion-based sensitivity analyses

| **Outcome** | **Sensitivity scenario** | **Studies** | **Participants (DEX/control)** | **Effect (95% CI)** | **P value** | **I2** |
| --- | --- | --- | --- | --- | --- | --- |
| POD incidence | Excluding high-risk-of-bias trial (Gao 2021) | 14 | 1525/1592 | RR 0.72 (0.59 to 0.88) | 0.001 | 13.8% |
| POD incidence | Excluding factorial trial (Subramaniam 2019) | 14 | 1515/1582 | RR 0.69 (0.55 to 0.85) | <0.001 | 23.6% |
| Delirium duration | Excluding factorial trial (Subramaniam 2019) | 5 | 79/118 | MD -1.15 (-2.17 to -0.12) | 0.028 | 86.1% |
| ICU length of stay | Excluding high-risk-of-bias trial (Gao 2021) | 8 | 1056/1067 | MD -0.20 (-0.72 to 0.32) | 0.447 | 54.2% |
| ICU length of stay | Excluding factorial trial (Subramaniam 2019) | 8 | 1046/1057 | MD -0.39 (-0.78 to -0.01) | 0.046 | 35.7% |
| ICU length of stay | Excluding studies converted from median/IQR | 5 | 376/369 | MD -0.38 (-1.02 to 0.25) | 0.232 | 70.9% |
| Hospital length of stay | Excluding high-risk-of-bias trial (Gao 2021) | 7 | 1026/1037 | MD 0.00 (-1.07 to 1.07) | 0.998 | 96.9% |
| Hospital length of stay | Excluding factorial trial (Subramaniam 2019) | 7 | 1016/1027 | MD -0.49 (-1.66 to 0.69) | 0.416 | 97.6% |
| Hospital length of stay | Excluding studies converted from median/IQR | 4 | 346/339 | MD -1.22 (-2.71 to 0.28) | 0.110 | 92.6% |
| Bradycardia | Excluding factorial trial (Subramaniam 2019) | 5 | 791/804 | RR 2.05 (1.31 to 3.20) | 0.002 | 0.0% |
| Hypotension | Excluding factorial trial (Subramaniam 2019) | 5 | 791/804 | RR 0.90 (0.55 to 1.48) | 0.678 | 58.7% |

Table 3: Comparison of primary DerSimonian-Laird random-effects analyses with Hartung-Knapp-Sidik-Jonkman sensitivity analyses

| **Outcome** | **Primary analyses** | **P value** | **HKSJ random-effects** | **HKSJ P value** | **I2 (%)** |
| --- | --- | --- | --- | --- | --- |
| **Incidence of delirium** | 0.70 (0.56 to 0.86) | 0.001 | 0.70 (0.56 to 0.87) | 0.004 | 19 |
| **Duration of delirium** | -1.12 (-1.89 to -0.35) | 0.01 | -1.12 (-2.06 to -0.19) | 0.027 | 83 |
| **Length of ICU stay** | -0.26 (-0.67 to 0.17) | 0.21 | -0.26 (-0.72 to 0.21) | 0.239 | 49.2 |
| **Length of hospital stay** | -0.37 (-1.39 to 0.65) | 0.51 | -0.37 (-1.58 to 0.84) | 0.49 | 97.2 |
| **Bradycardia** | 2.08 (1.31 to 3.31) | 0.0019 | 2.08 (1.32 to 3.30) | 0.009 | 0 |
| **Hypotension** | 1.09 (0.64 to 1.83) | 0.76 | 1.09 (0.48 to 2.44) | 0.805 | 68 |
