# Supplementary material for: Efficacy and hemodynamic safety of perioperative dexmedetomidine for the prevention of postoperative delirium in elderly patients undergoing cardiac surgery: an updated systematic review and meta-analysis of randomized controlled trials
Source: Front Neurol. 2026 Aug 12;17:1895312. doi: 10.3389/fneur.2026.1895312 (PMC13506365; doi:10.3389/fneur.2026.1895312)
Supplement: Supplementary file 4 [file Supplementary_file_4.DOCX]

**Supplementary Material 4:** GRADE Summary of Findings

| **Outcome** | **Studies / participants** | **Effect estimate** | **Certainty** | **Main reasons for downgrading** |
| --- | --- | --- | --- | --- |
| Incidence of POD | 15 RCTs / 3,157 participants | RR 0.70 (95% CI 0.56 to 0.86) | Moderate | Downgraded for indirectness due to comparator and clinical heterogeneity. |
| POD duration | 6 RCTs / 214 participants with POD | MD -1.12 days (95% CI -1.89 to -0.35) | Low | Downgraded for serious inconsistency (I2=83%) and imprecision/small contributing sample. |
| Length of ICU stay | 9 RCTs / 2,163 participants | MD -0.26 days (95% CI -0.68 to 0.17) | Low | Downgraded for inconsistency and imprecision; no statistically significant reduction. |
| Length of hospital stay | 8 RCTs / 2,103 participants | MD -0.37 days (95% CI -1.39 to 0.65) | Very low | Downgraded for very serious inconsistency (I2 approximately 97%), imprecision, and possible publication bias. |
| Bradycardia | 6 RCTs / 1,655 participants | RR 2.08 (95% CI 1.31 to 3.31) | Low | Downgraded for indirectness because event definitions and clinical severity/interventions were inconsistently reported. |
| Hypotension | 6 RCTs / 1,655 participants | RR 1.09 (95% CI 0.64 to 1.83) | Low | Downgraded for inconsistency, imprecision, and heterogeneous definitions of hypotension. |
